# Sacral Erector Spinae Plane Block for Gender Reassignment Surgery

**DOI:** 10.7759/cureus.7665

**Published:** 2020-04-14

**Authors:** Promil Kukreja, Paige Deichmann, John P Selph, John Hebbard, Hari Kalagara

**Affiliations:** 1 Anesthesiology and Perioperative Medicine, University of Alabama at Birmingham, Birmingham, USA; 2 Urology, University of Alabama at Birmingham, Birmingham, USA

**Keywords:** erector spinae plane block, gender reassignment surgery, transgender, truncal block, regional anesthesia, interfascial plane block, penectomy, multimodal analgesia, esp

## Abstract

The erector spinae plane block (ESPB) is an interfascial plane block that has been used to provide perioperative analgesia for a variety of indications. This case report describes the novel use of the sacral ESPB on a transgender patient undergoing male-to-female gender reassignment surgery for perioperative pain control. The sacral ESPB technique was described and post-operative pain score and opioid requirements were reported. The sacral ESPB was successfully used as an alternative to neuraxial, caudal, or peripheral nerve blocks for gender reassignment surgery.

## Introduction

The erector spinae plane block (ESPB) is a novel paraspinal plane block first described in 2016 to relieve thoracic pain [1]. Since that time, the ESPB has been used for a number of novel indications and has been described in over 200 case reports [2]. Randomized controlled trials have shown the ESPB to be an effective perioperative analgesic for several thoracic procedures, including mastectomy, video-assisted thoracoscopy (VATS), and cardiac surgery [3-6]. At our institution, we regularly utilize the thoracic ESPB as a component of our regional anesthesia pain service for both chest and abdominal surgeries. More recently, the lumbar ESPB has been described for abdominal surgeries, prostatectomy, lumbar spine surgery, total hip arthroplasty, and proximal femur surgery [7-12].

Tulgar et al. were the first to describe the sacral ESPB as a means to provide analgesia to sacral dermatomes for a pilonidal sinus surgery [12].

After reading the aforementioned sacral ESPB case reports, we proceeded with the sacral ESPB for a novel indication. Using the midline approach described by Aksu et al., we performed the sacral ESPB on a patient undergoing gender reassignment surgery for perioperative pain control [13]. The sacral ESPB has since been described to provide pain control for pediatric hypospadias repair and lower extremity radicular pain [14,15].

The number of individuals in the US who carry a diagnosis of either gender identity disorder or transsexual has increased by over three fold from 2000 to 2014 and continues to increase [16]. Of those individuals, approximately 20%-40% seek gender reassignment surgery [16]. The incidence of gender reassignment surgery will likely rise as societal barriers, such as social stigma, access to care, and insurance coverage, are overcome. As these procedures become more common, anesthesiologists should seek out modalities to address perioperative pain.

## Case presentation

A 39-year-old American Society of Anesthesiologists (ASA) 2 transwoman, with a weight of 98.9 kg and height of 177 cm (body mass index (BMI) 31.3 kg/m2), presented for gender reassignment surgery. Patient's gender reassignment surgery would include a total penectomy, bilateral orchiectomy, complete scrotectomy, anterior urethroplasty, construction of artificial vagina with graft, perineoplasty, clitoroplasty, and cystoscopy. A preoperative sacral ESPB for pain control was planned as part of a multimodal pain regimen. The morning of the surgery, the patient was placed in prone position with standard monitoring and given 1 mg midazolam and 50 mcg fentanyl for sedation. Under aspetic precautions, a high-frequency linear ultrasound transducer was placed midline just above the sacrum. 

**Figure 1:**
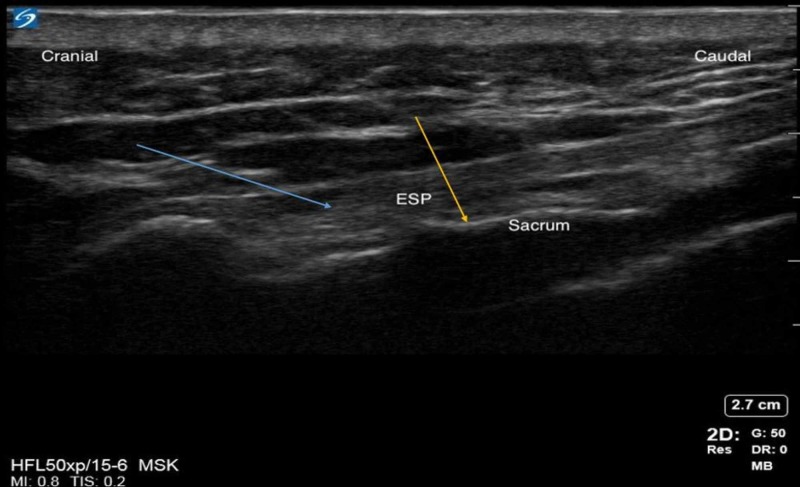
Sacral erector spinae plane Blue arrow indicates the erector spinae plane. Yellow arrow indicates the sacrum.

**Figure 2:**
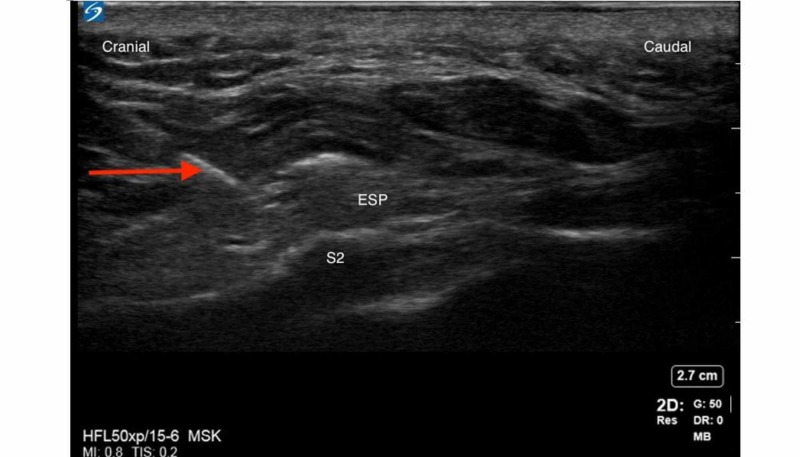
S2 median sacral crest and needle path (red arrow) The red arrow indicates the needle.

Median sacral crests and erector spinae muscle/plane (ESP) were identified as described by Aksu et al. A 5 cm, 21G echogenic needle was inserted using an in-plane technique from cranial to caudal direction and advanced to the S4 crest (Figure 1). Following negative aspiration, 20 mL of 0.2% ropivacaine was injected at the S4 median sacral crest level with aspiration every 5 mLs. The process was repeated at the S2 median sacral crest level with an additional 20 mL of 0.2% ropivacaine (Figure 2). Local anesthetic (LA) was deposited in the appropriate fascial plane between the erector spinae muscles and the S4 and S2 median sacral crests. 

**Figure 3:**
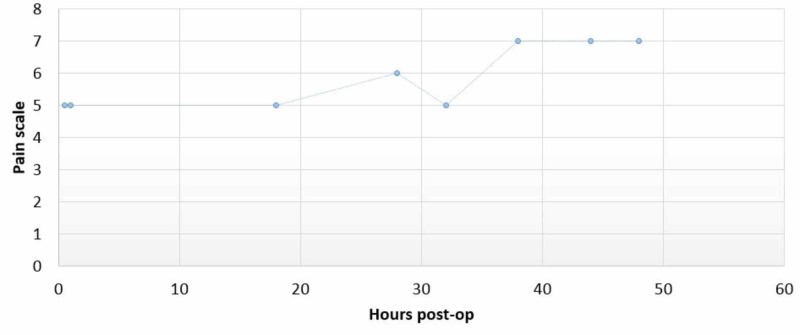
Pain score over time

The patient was then taken to the operating room for surgery under general anesthesia. The surgery lasted six hours and 20 minutes with total intraoperative opioid consumption of 150 mcg of fentanyl for the entire procedure. After surgery, the patient was transported to post-anesthesia care unit (PACU) where she rated her post-operative pain as moderate (numerical rating scale 4-6). The post-operative pain scores are shown in Figure 3.

**Figure 4:**
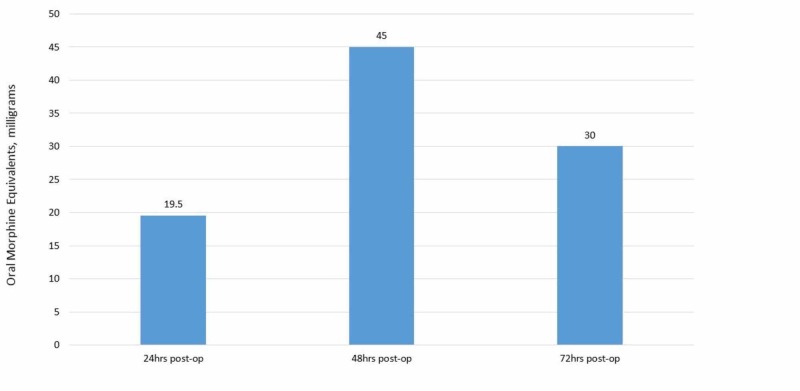
Post-operative oral morphine equivalents (OMEs) over time

Patient's complete post-operative pain regimen consisted of scheduled gabapentin 100 mg TID, acetaminophen 650 mg q6, ibuprofen 600 mg TID and PRN oxycodone 5-10 mg and PRN IV hydromorphone 0.2 mg for breakthrough pain. Daily oral morphine equivalents (OMEs) are listed below in Figure 4. Over the course of her hospitalization, she never required IV hydromorphone for breakthrough pain control.

## Discussion

Gender-reassignment surgery was a challenging case for peri-operative analgesia to effectively control pain. There is evidence to support the use of neuraxial blocks to control post-operative pain, but we had some reservations based on the associated adverse effects with neuraxial blocks like hypotension. Also, post-operative anticoagulation regimen after neuraxial block may increase the risk of rare but dangerous adverse effects such as epidural hematoma. The location of the surgery and expected area of pain distribution precluded the consideration of transversus abdominis plane (TAP), quadratus lumborum, or lumbar plexus blocks. Pudendal nerve blocks would have enabled analgesia, but the risk of nerve injury due to close approximation to the surgical site and the expertise of regional anesthesia staff being comfortable in doing these bilateral pudendal nerve blocks moved us away from this option. Also, these nerve blocks would not have covered the affected area completely if used individually. As an alternative, we decided to proceed with a sacral ESP block. 

ESPB is a relatively new regional interfacial plane block that provides somatic and visceral analgesia blocking the dorsal and ventral rami of spinal nerve along with the rami communicantes that transmit autonomic fibers to and from the sympathetic ganglia. Branches of the dorsal ramus innervates the skin of the back and branches of the ventral ramus supply lateral and anterior wall. ESPB has extensive cranio-caudal spread of LA and helpful in blocking multiple dermatomes [16].

ESPB has potential applications where risk of using epidural outweighs benefits. ESPB block at sacral levels can potentially block pudendal nerve (S2-S4), and may also block part of lumbar plexus via cephalad spread. There is no other individual nerve block besides neuraxial block that can cover both perineum and genitalia. The innervation of external genitalia is mainly by pudendal nerve which arises from S2-S4 levels and accompany the pudendal vessels. Two more nerves ilioinguinal (L1) and genital branch of genitofemoral nerve (L1-L2) arise from lumbar plexus to innervate regions of external genitalia. The sacral ESPB offers a practical alternative to neuraxial block or caudal block to provide analgesia for such urological procedure.

The patient in this case report received very minimal opioids intraoperatively and also in the early post-operative period along with moderate pain scores until the block effect wore off. The midline technique offers single injection rather than transverse approach which requires bilateral injections to perform sacral ESP block. We preferred to do sacral ESP at 2 levels in this case at S4 and S2 levels with the aim of covering the pudendal innervation from S4 injection and to cover lower lumbar levels from S2 injection. The Sacral ESP block has not been described before for urological procedures like gender re-assignment. The ESP block has shown to effectively treat chronic thoracic pain, acute post-thoracotomy pain, breast surgery, as well as providing abdominal analgesia. There is minimal literature describing the use of ESP block for adult urological procedures, although there is some evidence for pediatric urological procedures. This is a novel technique described for the first time for these gender reassignment surgeries using a 2 level sacral ESP block. Most of these patients undergo repeated minor interventions in the early postoperative period which are quite painful and the potential for sacral ESP catheter to enable prolonged analgesia during the hospital stay is another likely advantage with this block in these surgeries.

The limitations are this is just one case report and we need further studies to validate the analgesic benefits of sacral ESP for these surgeries. We could not assess the sensory distribution of the block due to time constraints and there was no lower extremity motor weakness in the post-operative period. There is no clarity over the anatomical nomenclature for these sacral interfascial plane blocks currently as suggested by Hamilton et al. and proposed for sacral retrolaminar block as an alternative name [17]. Piracinni et al. proposed the name of sacral multifidus plane block instead of retrolaminar or ESP nomenclature [13,14,18-20]. We suggest using any of the above nomenclatures as each of them are identical but with different proposed anatomical names. The sacral ESP block can offer many advantages, which were relevant to our patient. First, it provides coverage of multiple dermatomal levels by a longitudinal midline injection technique. Second, it is a superficial block and can be safely performed under ultrasound guidance. Third, it does not cause significant motor weakness. Fourth, ESP block may not cause significant hypotension and more hemodynamically stable compared to neuraxial blocks. Fifth, the potential to place the sacral ESP catheter to provide prolonged post-operative analgesia. These unique characteristics clearly favored sacral ESP block over other blocks like neuraxial, caudal, lumbar plexus, quadratus lumborum or pudendal nerve blocks

## Conclusions

The sacral ESP (sacral retrolaminar or sacral multifidus plane) block is an alternative regional analgesic technique for urological procedures including gender reassignment procedures. It can be used as a viable alternative to neuraxial, caudal blocks and peripheral nerve blocks (pudendal nerve block) for these unique surgeries in this patient population. The efficacy of the sacral ESP blocks is not well established yet, randomized controlled trials are warranted for better understanding of the sacral ESP blocks with respect to analgesic efficacy and adverse effects. There needs to be a consensus on the nomenclature going forward for these sacral interfascial plane blocks to be adopted universally.
